# Effectiveness of Transcranial Direct Current Stimulation (tDCS) during a Virtual Reality Task in Women with Fibromyalgia—A Randomized Clinical Study

**DOI:** 10.3390/brainsci14090928

**Published:** 2024-09-18

**Authors:** Thaís Nogueira da Silva, Vivian Finotti Ribeiro, Margot Carol Condori Apaza, Lívia Gallerani Romana, Íbis Ariana Peña de Moraes, Eduardo Dati Dias, Suely Steinschreiber Roizenblatt, Juliana Perez Martinez, Fernando Henrique Magalhães, Marcelo Massa, Alessandro Hervaldo Nicolai Ré, Luciano Vieira de Araújo, Talita Dias da Silva-Magalhães, Carlos Bandeira de Mello Monteiro

**Affiliations:** 1 Graduate Program in Rehabilitation Sciences, Faculty of Medicine, University of São Paulo (FMUSP), São Paulo, Brazil; 2 Graduate Program in Medicine (Cardiology), Escola Paulista de Medicina, Federal University of São Paulo (EPM/UNIFESP), São Paulo, Brazil; 3 Graduate Program in Physical Activity Sciences, School of Arts, Science and Humanities, University of São Paulo (EACH-USP), São Paulo, Brazil; 4 Department of Medicine, University of City of São Paulo (UNICID), São Paulo, Brazil; 5 Department of Physical Therapy, Faculty of Sciences and Technology (FCT/UNESP), State University of São Paulo, São Paulo, Brazil; 6 Graduate Program in Bioengineering, University Brazil, São Paulo, Brazil

**Keywords:** fibromyalgia, transcranial direct current stimulation, virtual reality, perceptual performance, motor performance, rehabilitation

## Abstract

Background/Objectives: Fibromyalgia (FM) is a chronic condition characterized by widespread musculoskeletal pain, fatigue, and impaired motor performance. This study aimed to investigate the effects of transcranial direct current stimulation (tDCS) during virtual reality (VR) tasks on the motor performance of women with FM. Methods: Participants were divided into two groups: Group A received active tDCS for 10 days followed by sham tDCS for 10 days, while Group B received the opposite sequence. Both groups performed VR tasks using MoveHero software (v. 2.4) during the tDCS sessions. Motor performance was assessed by the number of hits (movement with correct timing to reach the targets) and absolute (accuracy measure) and variable (precision measure) errors during VR tasks. Participants were 21 women, aged 30–50 years, and diagnosed with FM. Results: Group A, which received active tDCS first, presented significant improvements in motor performance (number of hits and absolute and variable errors). The benefits of active tDCS persisted into the sham phase, suggesting a lasting neuroplastic effect. Conclusions: tDCS during VR tasks significantly improved motor performance in women with FM, particularly in complex, extensive movements. These findings indicate that tDCS enhances neuroplasticity, leading to sustained motor improvements, making it a promising therapeutic tool in FM rehabilitation.

## 1. Introduction

Fibromyalgia (FM) is recognized as a central sensitivity syndrome, characterized by central sensitization—a process where neuronal signal amplification within the central nervous system heightens pain perception [1]. This phenomenon has been linked to immunological mechanisms, neuroinflammation, and the involvement of peripheral nerves, with potential biomarkers such as microRNAs (miRNAs) offering further insight into its underlying pathophysiology [2]. FM is a poly-symptomatic distress syndrome that often overlaps with similar disorders and is not an exclusion diagnosis, as it can co-exist with other clinical conditions. Clinically, it is marked by chronic widespread pain, fatigue, sleep disorders, and cognitive disturbances, with symptoms that can fluctuate significantly [3,4].

Although all of these symptoms can affect quality of life for women with FM [5], motor performance difficulties have a significant impact on daily life. FM can result in reduced strength and decreased endurance, leading to impaired coordination, making it challenging for people with this condition to perform daily activities and engage in physical exercise, due to a fear of movement and avoidance behaviors [6,7]. Thus, regular physical exercise is crucial for FM patients, as it helps to maintain muscle strength, improve cardiovascular health, and enhance overall quality of life [8,9].

Given the critical need for innovative rehabilitation strategies that enhance motor function in fibromyalgia (FM), transcranial direct current stimulation (tDCS) has emerged as a promising intervention. Evidence is increasing on the effectiveness of this non-invasive neuromodulation technique for improving motor performance and overall function in FM patients, offering a novel approach to addressing the multifaceted challenges of this condition [10].

tDCS operates by applying low-intensity currents through electrodes placed on the scalp, inducing alterations in neuronal firing rates and synaptic plasticity. This process promotes neuroplasticity in key cortical areas [11,12], which is crucial for enhancing motor learning and long-term potentiation. By modulating synaptic connections, tDCS provides a physiological basis for the functional improvements observed in FM patients, supporting its growing role as an effective therapeutic tool [13].

Enhancing sensorimotor and physiological functions, tDCS has shown efficacy in various clinical disorders by altering brain activity [14,15]. Research highlights significant improvements in functionality and mobility in patients with conditions such as Parkinson’s Disease [16], spinal cord injury [17], and stroke [18,19]. Furthermore, Kesikburun (2022) [20] demonstrated that tDCS facilitates motor recovery and enhances performance in daily activities for individuals suffering from stroke, spinal cord injury, traumatic brain injury, and multiple sclerosis.

Thus, tDCS plays a crucial role in facilitating physical activity for individuals with fibromyalgia, by reducing symptoms such as pain and fatigue [10] and helping to alleviate the fear of movement and avoidance behaviors that often prevent patients from participating in regular exercise [7]. This makes tDCS a promising tool in fibromyalgia management, as it supports patients in maintaining muscle strength, cardiovascular health, and an improved quality of life through exercise [10].

Some studies have investigated the effects of tDCS on motor function and pain. Khedr et al. (2017) [21] found that the use of tDCS for 10 sessions can reduce pain levels. Similar results were observed by Fagerlund et al. (2015) [22] and Fregni et al. (2006) [23] after five sessions of tDCS. Long-term results are associated with extended treatment periods, as shown by studies employing tDCS for 10 sessions [21,24]. Furthermore, systematic reviews [25,26] and a meta-analysis [27] strengthen these findings by highlighting functional improvements after tDCS in FM patients.

To effectively evaluate motor improvements in fibromyalgia patients undergoing tDCS treatment, it is important to utilize motor tasks that offer consistent conditions for an accurate assessment and sustained patient engagement. Thus, Virtual Reality (VR) offers an innovative solution, creating controlled environments that ensure consistent conditions while also boosting patient interaction and motivation.

Studies on individuals with FM have shown the beneficial effects of VR in inducing positive emotions, alleviating depression [28,29], enhancing daily activity performance, and reducing the severity of symptoms, such as pain [30,31].

Applying tDCS during VR interventions can amplify rehabilitation outcomes by enhancing neural plasticity and motor learning, making this approach particularly promising for improving motor performance and quality of life in FM patients [32]. The application of tDCS during VR tasks engages both cognitive and motor networks, offering a multidimensional therapeutic strategy for FM.

Considering these positive findings, the current study aimed to evaluate the impact of tDCS therapy during VR tasks on performance improvement in FM patients. As noted by Cheng et al. (2023) [33], identifying the benefits of electrical neuromodulation alongside other interventions is crucial for advancing FM treatment. We hypothesized that FM patients would experience enhanced motor performance during the VR task, with greater improvements observed during the active tDCS phase. If confirmed, these results will significantly contribute to improved FM treatment strategies.

## 2. Materials and Methods

### 2.1. Study Design and Location

This is a longitudinal, crossover, randomized, controlled clinical trial. Trial registration number:BRB-2nt4byr—Effect of Neuromodulation and Virtual Game on Cardiac Variation in Women with Fibromyalgia, registration date: 23 May 2024, this registration was done retrospectively. This study was approved by the Research Ethics Committee of the University of São Paulo (CAAE: 56691722.9.0000.0068) and complied with Resolution 466/2012 of the National Health Council of 10 October 1996, which regulates research involving human beings, and the Declaration of Helsinki (1964).

All volunteers were informed about the objectives and procedures of the study and, when they agreed, they signed the Free and Informed Consent Form. The data were stored electronically in databases with restricted and secure access. All data were encoded with the removal of any information that would make it possible to identify the individuals involved.

### 2.2. Participants

The current study included women with a diagnosis of fibromyalgia, confirmed by a medical specialist, according to the Fibromyalgia diagnosis criteria of the American College of Rheumatology [34]; aged between 30 and 50 years; and recruited through social media, after meeting the inclusion criteria.

A total of 86 volunteers were recruited to participate in the protocol. However, 65 volunteers were not included (61 because they did not attend the first evaluation and 4 preferred not to participate in the study), ending with a sample of 21 participants, randomized into two groups (GA: *n* = 11; GB: *n* = 10) according to the sequence performed. Table 1 presents the sample characterization data and the comparisons between the two groups evaluated, indicating that the groups were homogeneous in relation to the variables presented in the table, avoiding bias in the results in the different support bases. Table 2 presents the medications taken by the subjects of the study, in order to characterize our sample. Of the 21 participants, 8 (38%) did not take any medication, and although only one subject reported having a diagnosis of depression, 10 (47.6%) participants were using antidepressants.

### 2.3. Inclusion and Exclusion Criteria

As inclusion criteria, we considered: participants who agreed to participate in the study through informed consent and who presented; (1) were aged between 30–50 years; and (2) had a clinical diagnosis of fibromyalgia.

The following were considered as non-inclusion criteria: (1) recruited participants that not attend the first evaluation day; (2) recruited participants that decided to not be included in the protocol for specific reasons (before randomization); (3) diagnosis of cardiovascular, pulmonary, orthopedic, or other systemic diseases (such as cancer and diabetes) that could limit exercise practice [35]; and (4) epilepsy, metal implants, pregnancy, or scalp injury as tDCS contraindication [36].

As exclusion criteria, the following were considered: (1) individuals who did not understand or were unable to perform the proposed activities; (2) participants who could not be present on the day of the evaluation and the intervention (after randomization); and (3) individuals who withdrew from participating during the protocol.

### 2.4. Virtual Reality Intervention Task (Software MoveHero)

The volunteers performed the tasks individually, in a room with a computer, a table, and a chair, and in the presence of the responsible evaluator. Before starting the task, the researcher explained the game verbally and performed a demonstration of its operation, and then the participants performed a single attempt at the game while standing, to ensure they understood the instructions. For the virtual reality task, the game MoveHero was used.

The MoveHero software was developed at the School of Arts, Sciences and Humanities at the University of São Paulo and has been used in different studies, including research on cerebral palsy [37], autism spectrum disorder [38,39], and spinal cord injury [40].

**Figure 1:**
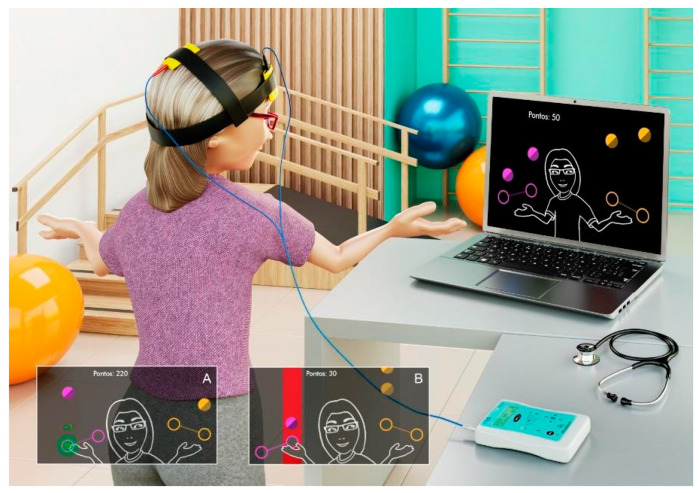
Illustrative picture of a participant playing the MoveHero game while receiving tDCS. (**A**) Example of target hit correctly, with green feedback. (**B**) Example of missing target, with red feedback.

The game presents four fixed targets and falling spheres on the computer screen, with a predefined speed and number of spheres based on a song that is chosen by the researcher. To successfully complete the game, the participant needs to intercept the spheres when they reach one of the four targets. The targets are in parallel, two to the left and two to the right of the volunteer and are denominated targets A, B, C, and D (from left to right) (Figure 1).

To conduct the game, a webcam is activated that captures the participant’s upper limb movements, from a position at least 0.5 to 1 m from the computer screen. The game offers hit and miss feedback by changing the color of the spheres (green for hit and red for miss), and the total score is visible in the upper left corner of the screen, with 10 points for each hit.

### 2.5. Anodal Transcranial Direct Current Stimulation

Anodal tDCS was applied for 20 min at an intensity of 2 mA (with a 20 s ramp of ascent and descent) over the scalp, as recommended by Bikson et al. (2016) [41], with the anode positioned in the cortical area corresponding to the primary motor cortex (C3), and the cathode positioned over the contralateral supraorbital area (Fp2 area), according to the 10/20 EEG system. Stimulation was performed using two electrodes, placed inside sponges (5 × 5 cm), moistened with saline solution and fixed at the target site for stimulation or referral [41,42].

In the sham stimulation, the same settings and parameters were used as in the active stimulation, but the current was applied for 20 s, only to induce the initial tingling sensation that some patients report experiencing during tDCS stimulation [41,43]. Then, over the following 1 min, this current was progressively turned off. This configuration ensures that the electrical stimulus is interrupted before generating any considerable effect, while the other characteristics of the intervention are maintained. Therefore, the participants feel the sensation of the stimulus, which guarantees blinding. The device used was a direct current stimulator (MicroEstim Foco Research, NKL^®^, Belo Horizonte, Brazil) that allows for the blinding of the research volunteers and the researchers.

### 2.6. Procedures

After meeting the inclusion criteria, the volunteers in the study were coded by a third researcher (for blinding the study) and then asked to complete a Sociodemographic Questionnaire. They were then divided into two groups: Group A—started with active tDCS settings before being changed to the sham settings; Group B: started with the sham tDCS settings and were then changed to the active settings, with a 1:1 allocation, defined using a site (randomization.com (accessed on 7 August 2024)), by an independent researcher who was not involved with the recruitment of participants or with the evaluations. The volunteers, researchers, and evaluators remained blind to the allocation of the groups. To ensure proper concealment, participants were given codes and separated from the allocation process by a different investigator. The researchers responsible for the application of the intervention and the outcome evaluators did not know the allocation of the participants. In addition to blind the experimenter, the device used has a “study” mode, in which each participant’s code was entered (code given by the independent researcher), so that the device (MicroEstim Foco Research, NKL^®^) recognized and programmed the settings independently (active or sham).

After the random assignment of participants to the study groups, the sessions of anodic tDCS (either sham or active) with virtual tasks were administered on weekdays. Following the first 10 sessions of the intervention, participants underwent a 1-month washout period. After this period, the groups were crossed over, and participants received an additional 10 sessions of anodic tDCS (either sham or active) with virtual tasks.

Initially, the consent forms were presented to the participants, as well as the procedures and objectives of the study. After the participant had signed, the following information was collected to characterize the sample: age, height, weight, and time of diagnosis (in years).

After the data collection, the volunteers were seated in a normal chair, with their hands placed on their legs and feet flat on the floor. Next, the electrodes were demarcated and placed according to the International 10–20 system for EEG. The volunteers then adopted a standing position, and the application of tDCS was initiated, while they simultaneously performed training with virtual tasks for 10 min. The task used was the virtual game MoveHero, which had been previously explained and demonstrated by the researcher.

**Figure 2:**
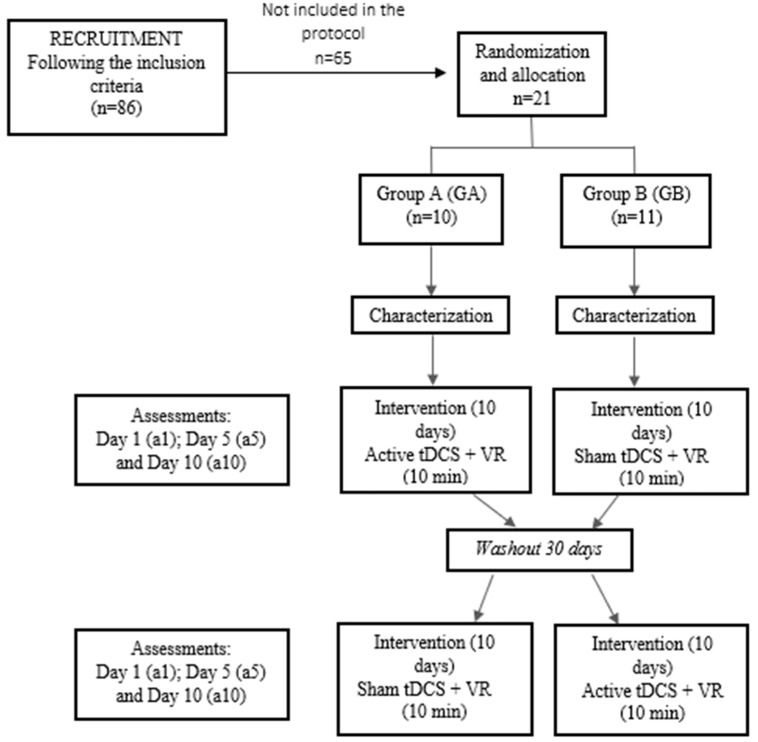
Flowchart of the study procedures.

For the long-term evaluation, assessments were conducted on the 1st (a1), 5th (a5), and 10th (a10) days of the intervention, during both the first period and, following the washout phase, the second period (Figure 2).

### 2.7. Assessments

To assess the participants’ performance, error measures were considered, defined as the difference between the moment the sphere hit the target (arrival time) and the time the touch or gesture was registered in milliseconds. The measures included: Absolute Error (AE), which demonstrates the accuracy of the movement; and Variable Error (VE), which identifies the precision of the movement [44,45]. The number of hits (balls hit on target) during the game was also recorded.

To analyze the performance, we divided the four targets (explained in Section 2.4) into central (targets B and C) and lateral (targets A and B) positions. According to Lin et al. (2023) [46], upper extremity motor control contains both proximal and distal elements. Proximal elements include shoulder strength and the ability to isolate movements, while distal elements include finger strength and individuation (i.e., the ability to precisely control individual fingers). In addition, there are distinct structural and functional organizations of circuits for proximal and distal motor control in the healthy central nervous system. Therefore, this analysis was carried out in order to assess and identify differences in performance during movements closer to the medial axis, i.e., central targets, and with the arms open, i.e., lateral targets. This separation could predict whether proximal and distal upper extremity segments were selectively impaired by neuromuscular conditions [46].

### 2.8. Statistical Analysis

To conduct data analysis, descriptive statistics were performed for sample characterization, and the results are presented as mean values, standard deviation, and dispersion values. For the independent variables of the volunteers, the Student’s *t*-test (for continuous variables) was used to compare the sham and active intervention groups. For the dependent variables in MoveHero, we used the Absolute Error (AE), Variable Error (VE), and hits for Central and Lateral positions.

Data were analyzed based on the intention-to-treat principle. The dependent variables were subjected to a Linear Mixed Model (LMM) with 2 (Groups: A-Active->Sham and B-Sham->Active) by 2 (Interventions: Active and Sham) by 3 factors (Assessments: a1, a5 and a10), with repeated measures on the last two factors.

The data in the graphs and the results are presented as mean (M) and standard error. The statistical package used was SPSS, version 26.0, and values of *p* < 0.05 were considered significant.

## 3. Results

### 3.1. Number of Hits

**Figure 3:**
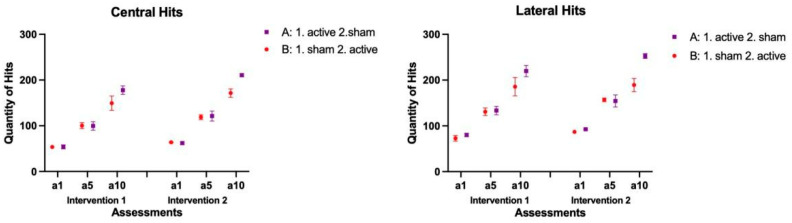
Representation of the mean and standard error of the Number of Hits in both Sequences and all assessments. A: Group A; B: Group B; a1: assessment day 1; a5: assessment day 5; a10: assessment day 10.

The mean and standard errors are presented in Figure 3. Considering the quantity of Hits, in the Central position, we found a main effect for the factors: Groups (F = 5.985; *p* = 0.016; Estimate = −39.2), Intervention (F = 16.777; *p* < 0.001; Estimate = −32.9), and Assessments (F = 224.173; *p* < 0.001; Estimate = −148.6), and an interaction between Group and Assessments (F = 5.878; *p* = 0.004; Estimate = 40.9). Regarding Groups, comparisons showed that group A (active–sham) achieved a higher number of hits (M = 120) than group B (sham–active; M = 109). Considering the main effect for Interventions, the participants presented a higher number of hits in the Second block of interventions (M = 124) than in the First block (M = 105). For Assessments, all participants demonstrated increases in the number of hits from a1 to a5, a5 to a10, and a1 to a10 in both blocks of interventions. When looking at the interaction between Groups and Assessments (and Interventions), the post-hoc test showed that Group A presented more hits than Group B in a10 in both blocks of intervention (Intervention 1: Group A:177.7, Group B: 149.4; *p* = 0.012|Intervention 2: Group A: 210.7, Group B: 171.5; *p* = 0.001).

Considering the Lateral position, we found a main effect for the factors Groups (F = 9.750; *p* = 0.002; Estimate = −63.4), Intervention (F = 9.659; *p* = 0.003; Estimate = −32.7), and Assessments (F = 160.466; *p* < 0.001; Estimate = −160.4), and an interaction between Groups and Assessments (F = 6.620; *p* = 0.002; Estimate = 57.8). Similarly to the Central Position, the main effect for Group showed that in group A (active-sham) the participants achieved a higher number of hits (M = 155) than group B (sham-active; M = 137). Considering the effect for Interventions, in both groups the participants achieved a higher number of hits in the Second block of interventions (M = 155) than in the First block (M = 137). Regarding Assessments, all participants presented increases in the quantity of hits from a1 to a5, a5 to a10, and a1 to a10 in both blocks of interventions. In the interaction between Groups and Assessments (and Interventions), the post-hoc test showed that Group A presented more hits than group B in a10 in both blocks of intervention (Intervention 1: Group A:219.9, Group B: 185.7; *p* = 0.017|Intervention 2: Group A: 252.6, Group B: 189.2; *p* < 0.001). The post hoc test also showed that for the comparisons between Assessments in both Interventions, no significant differences were found from a1 of Intervention 1 to a1 of Intervention 2, while for a5 and a10 there were significant differences between Intervention 1 (a5: 100.1; a10: 163.6, respectively) and Intervention 2 (a5: 120.1; a10: 191.1|a5: *p* = 0.015; a10 *p* = 0.001).

Finally, when comparing Central and Lateral performance, there were significant differences within positions for both groups, in both Intervention 1 [Group A (Central: M = 110.4|Lateral: M = 144.5; *p* < 0.001) and Group B (Central: M = 101.2|Lateral: M = 129.8; *p* < 0.001)] and Intervention 2 [Group A (Central: M = 126.1|Lateral: M F= 160.7; *p* < 0.001) and Group B (Central: M = 118.1|Lateral: M = 144.3; *p* < 0.001)], showing that in the Lateral position, the participants presented a significantly higher number of hits.

### 3.2. Absolute Error—AE (Accuracy)

**Figure 4:**
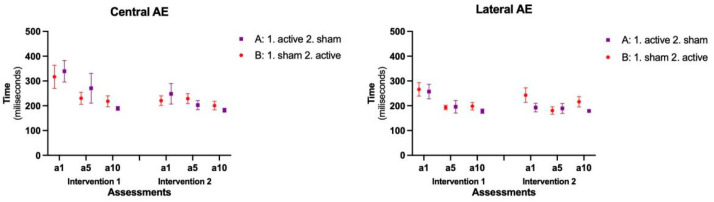
Representation of the mean and standard error of the Absolute Error (AE) in both Sequences and all assessments. A: Group A; B: Group B; a1: assessment day 1; a5: assessment day 5; a10: assessment day 10.

The mean and standard errors are presented in Figure 4. Furthermore, in the Absolute error, in the Central position, we found a main effect for the factors Intervention (F = 6.284; *p* = 0.014; Estimate = 7.1) and Assessments (F = 6.832; *p* = 0.002; Estimate = 66.2), and there was no interaction between the factors. For Interventions, comparisons showed a shorter error time in the Second intervention (M = 200 ms) than in the First intervention (M = 214 ms). Regarding Assessments, all participants presented decreases in AE from a1 to a5, a5 to a10, and a1 to a10 in both blocks of interventions. Although no interactions were found, the post hoc showed that for the comparisons between Assessments in both Interventions, a significant difference was found from a1 of Intervention 1 (M = 328.1) to a1 of Intervention 2 (M = 234.4; *p* = 0.004), with no significant difference between a5 and a10.

In the Lateral position, we found a main effect for the factor Assessments (F = 7.394; *p* = 0.001; Estimate = 13.5), that showed a higher error time in the first assessments in a1 participants (M = 239 ms) than in a5 (M = 189 ms; *p* = 0.001) and a10 (M = 192 ms; *p* = 0.002), except for Group A in Intervention 2. Although no significant interactions were found between Assessments and Interventions, the post-hoc test showed that only Group A presented a significant difference from a1 of Intervention 1 (M = 257.3) to a1 of Intervention 2 (M = 192.6; *p*= 0.030).

Lastly, when comparing Central to Lateral performance, there were significant differences within positions for both groups during Intervention 1 [Group A (Central: M = 266.5 ms|Lateral: M = 210.4 ms; *p* = 0.003) and Group B (Central: M = 254.9 ms|Lateral: M = 219.1 ms; *p* = 0.025)], while during Intervention 2, the significant difference occurred only for Group A [Group A (Central: M = 213.8 ms|Lateral: M = 187.2; *p* = 0.027)].

### 3.3. Variable Error (Precision)

**Figure 5:**
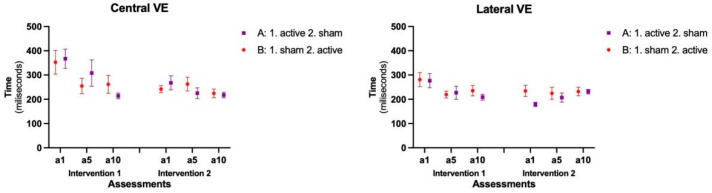
Representation of the mean and standard error of the Variable Error (VE) in both Sequences and all assessments. A: Group A; B: Group B; a1: assessment day 1; a5: assessment day 5; a10: assessment day 10.

The mean and standard errors are presented in Figure 5. For the Variable error, in the Central position, we found a main effect for the factors Intervention (F = 7.812; *p* = 0.006; Estimate = −3.7) and Assessments (F = 5.745; *p* = 0.004; Estimate = 50.1), while there was no interaction between the factors. Regarding Interventions, comparisons showed that in the Second intervention the participants had a shorter error time (M = 239 ms) than in the First intervention (M = 293 ms). For Assessments, although there was no interaction between factors, when considering the post-hoc tests, in the First block of intervention, we found a significant difference from a1 (M = 352.7 ms) to a5 (M = 254.9 ms; *p* = 0.030) and a1 to a10 (M = 261.5 ms; *p* = 0.043) in Group B, while for Group A the significant differences were between a1 (M = 367.1 ms) and a10 (M = 213.9 ms; *p* = 0.001), and between a5 (M = 308.1 ms; *p* = 0.046) and a10. In the second block of interventions, no significant differences were found between assessments. Furthermore, the post hoc test between Intervention and Assessments showed a difference from a1 in the first intervention to a1 in the second intervention block, with no significant difference for a5 and a10.

In the Lateral position, we found a marginal effect for the factor Intervention (F= 3.574; *p* = 0.061; Estimate = −22.8) and an interaction between Intervention and Assessments (F = 4.163; *p* = 0.018; Estimate = 42.9). For Interventions, comparisons showed that in the Second intervention the participants had a tendency to a shorter error time (M = 217 ms) than the First intervention (M = 241 ms). For the interaction between Intervention and Assessments (and Groups), in the first block of interventions, Group A presented a significant improvement in VE from a1 (M = 276.6 ms) to a10 (M = 208.5 ms; *p* = 0.027), while group B presented a significant improvement only from a1 (M = 281.0 ms) to a5 (M = 219.4 ms; *p* = 0.035). In the second block of intervention, no significant improvements were found. Furthermore, the post hoc test between Intervention and Assessments showed that there was a difference from a1 in the first intervention (M = 276 ms) to a1 in the second intervention (M = 178 ms; *p* = 0.002) only for Group A, with no significant difference for a5 and a10 in either group.

When comparing Central to Lateral performance, similarly to the absolute error, there were significant differences within position for both groups during Intervention 1 [Group A (Central: M = 296.3 ms|Lateral: M = 237.3 ms; *p* < 0.001) and Group B (Central: M = 289.7 ms|ateral: M = 245.2 ms; *p* = 0.009)], however, during Intervention 2, the significant difference occurred only for Group A [Group A (Central: M = 239.2 ms|Lateral: M = 203.6; *p* = 0.007)].

## 4. Discussion

The current study investigated the effectiveness of transcranial direct current stimulation (tDCS) during a virtual reality task, focusing on motor performance as measured by the number of hits (movement with correct timing to reach the targets), and absolute (accuracy measure) and variable (precision measure) errors. Our hypothesis was partially confirmed: although all participants demonstrated improved performance during the practice of the VR task, group A (active-sham) presented a better performance at different points in the variables analyzed. These results will be discussed below.

### 4.1. Improvements in Motor Performance with tDCS

The findings of the current study revealed that participants who received active tDCS first (Group A) showed notable improvements in motor performance. Specifically, they achieved a higher number of hits and exhibited reduced absolute and variable errors. This suggests that starting with active tDCS significantly enhances motor performance compared to starting with sham tDCS (Group B).

The crossover design, in which participants underwent both active and sham tDCS, offered valuable insights. A particularly intriguing result was Group A’s performance on the final assessment day (a10) of both intervention blocks. While improved performance in a10 of the first block (active tDCS) was anticipated, the sustained high performance during the second block (sham tDCS) suggests that the benefits of active tDCS persisted, potentially through enhanced motor performance.

This carryover effect (i.e., the effect of one intervention persisting and affecting the result of the subsequent intervention) indicates that active tDCS may induce lasting neuroplastic alterations, contributing to continued improvements even without ongoing stimulation [47]. These changes likely improved motor coordination and reduced errors, which were maintained when additional therapies, such as VR, were applied. The initial active tDCS phase appears to play a critical role in inducing lasting benefits, further supported by evidence showing that active tDCS improves functioning through enhanced neuroplasticity [33].

The enhanced motor performance observed in the group who received active tDCS first can be attributed to two key factors. Firstly, the application of tDCS over the primary motor cortex (M1) likely facilitated increased cortical excitability and neuroplasticity. According to Nitsche and Paulus (2000) [48] and Nitsche et al. (2003) [11], anodal stimulation can increase cortical excitability, promoting plasticity in the brain area. This neurophysiological alteration is critical for improving performance and execution, allowing participants to perform VR tasks with greater efficiency [49]).

Secondly, active tDCS at M1 regulates the cortical excitability, positively influencing fatigue and pain reduction [33,50] (Cheng et al., 2023, Azarkolah et al., 2023), and these benefits can contribute to the improvement in performance regarding the number of hits and errors (absolute and variable). These factors are crucial for successfully completing VR tasks, as they enable participants to maintain focus, process information quickly, and make decisions in real-time. According to Cheng et al. (2023) [33], the reduction in pain symptoms caused by neuromodulation can lead to a better quality of life and wellbeing, resulting in improvements in functioning.

### 4.2. Central and Lateral Target Positions

We designed the statistical analysis according to tasks divided into central and lateral target positions, in order to examine potential differences in motor performance, particularly focusing on the varying ranges of motion required for shoulder and elbow movements. Lateral targets involve broader, more extensive movements of the upper limbs, especially including hand and finger strength and the ability to control the movement, while central targets require smaller, more controlled motions closer to the body’s midline, involving more shoulder strength and the ability to isolate the movement [46]. This distinction may influence motor performance, which can be important for the rehabilitation of patients with Fibromyalgia.

When considering the number of hits, there was no difference in performance between central and lateral positions. However, for absolute error (which measures the difference between target time and actual response time—accuracy) some differences were found. Group A, which received active tDCS in the first intervention block, showed a better maintenance of performance in the lateral position during the subsequent sham phase. This group displayed a lower absolute error and the maintenance of improvements, suggesting that active tDCS had a lasting effect, particularly for tasks requiring more extensive movements. In contrast, this carryover effect was not observed in the central position.

On the other hand, for variable error (which measures response consistency and precision), both groups demonstrated improvements in the central position tasks during the active tDCS phase, while, in the lateral position, group A showed improvement only in the first intervention block, and group B’s progress plateaued in the second block.

These results suggest that, with the use of tDCS, performance in lateral targets was generally better than in central targets. Therefore, we can only speculate that:

(1) The diminished strength and fear of movement commonly observed in fibromyalgia may impair proximal motor performance, as these tasks require more complex, isolated movements. According to Nijs et al. (2013) [6] and Lavín-Pérez et al. (2024) [7] the fear of movement and avoidance behavior are prevalent in fibromyalgia patients due to pain and discomfort, leading to reduced engagement in motor tasks. This fear may particularly affect proximal movements, which involve larger muscle groups and more coordinated control, as they demand greater shoulder strength and precision. Furthermore, Bennett et al. (2009) [3] have noted that fibromyalgia often results in decreased muscle strength, which could further limit performance in tasks requiring proximal motor control, where muscle recruitment and fine coordination are essential.
(2) Fibromyalgia patients might find movements that require less range of motion, such as those involving proximal targets, more challenging. Sensory alterations in fibromyalgia, such as amplified sensory information and disrupted somatosensory processing, may affect motor control, particularly in tasks requiring smaller, more controlled movements. According to Bardal et al. (2016) [51], who investigated the micro movements of the upper limb in fibromyalgia, these sensory deficits might lead to greater reliance on visual feedback and low-frequency limb oscillations for upper limb control, potentially impairing proximal movements. Additionally, the mechanical properties of the limb could further influence micro-movements, highlighting the complex interaction between neural and mechanical factors in fibromyalgia patients.

### 4.3. The Importance of Repeated Practice of the VR Task during tDCS

Although tDCS contributed to performance improvements, these gains were primarily supported by the practice of VR tasks. The use of active tDCS during repeated engagement with VR tasks facilitated neuroplastic alterations that enhanced motor performance [32].

Repeated practice in VR tasks provides adaptation to the demands by creating and reinforcing neural connections [52]. This adaptation process leads to more accurate and consistent motor responses over time, as evidenced by the significant reductions in all variables analyzed in the study. The neuroplastic alterations promoted by VR tasks support the development of precise and reliable motor skills, essential for activities requiring fine motor control and coordination [52,53]. This continuous interaction and the immediate feedback provided by these VR tasks help to reinforce positive learning experiences, making it easier for participants to stay on track and progress steadily in their rehabilitation efforts. Consequently, VR task practice has been proven to be a highly effective method for enhancing motor performance, even when tDCS does not yield significant results.

Additionally, VR tasks offer real-time feedback, enabling participants to correct errors immediately. This immediate feedback loop allows for the fine-tuning of motor responses, resulting in enhanced precision, accuracy, and the number of hits. Participants can instantly see the outcomes of their actions, and make the necessary adjustments to improve progressively. Continuous feedback is a crucial element for enhancing performance and is effectively employed in VR settings to achieve substantial improvements in motor skills. According to Crocetta et al. (2018) [54], virtual reality provides three-dimensional artificial sensory feedback, whereby the user engages in experiences similar to real-life activities, promoting motor improvement. Furthermore, individuals with various disabilities and conditions can benefit from using MoveHero software, where an improvement in performance was seen during the VR game [39,55,56,57].

Moreover, interactive virtual reality using exergames may promote distraction from pain, which could contribute to a positive performance. A systematic review about the effect of interactive virtual reality on pain perception [58] reported the possibility of a positive influence of distraction using VR games on improvement in performance in patients with pain (including neck pain, phantom limb pain, chronic back pain, neuropathic pain, ankylosing spondylitis, post-mastectomy, subacromial impingement syndrome, and scapular dyskinesis).

According to Cortés-Pérez et al. (2021) [59], in a systematic review with a meta-analysis on the ability of VR to reduce the disabling impact of fibromyalgia, studies show that VR effectively enhances distraction, preventing patients from focusing on their fatigue and promoting better performance during their practice of the game.

### 4.4. Practical Implications for Rehabilitation

This study provides interesting insights into the potential benefits of the use of tDCS with VR tasks to enhance motor performance in individuals with fibromyalgia. The findings suggest that active tDCS, particularly when applied first, can induce lasting improvements in motor function, as seen in the increased number of hits and reductions in both absolute and variable errors. The enhanced performance, especially in tasks requiring broader, more complex movements (such as lateral targets), indicates that tDCS may help overcome motor impairments associated with fibromyalgia, putatively by promoting neuroplasticity and improving coordination. Importantly, the study shows that VR task practice can lead to sustained motor improvements, even when the tDCS effects are minimal, making it a valuable tool in addressing motor impairments in fibromyalgia patients.

### 4.5. Limitations

The current study presents several limitations that should be mentioned: (1) The small sample size, combined with the exclusive focus on women aged 30–50, limits the generalizability of the findings and reduces the overall reliability of the results. (2) Due to the small sample size and the number of variables, it was not possible to analyze the interaction of medication and our proposed intervention. While there is currently no evidence suggesting that the combination of pharmacotherapy with tDCS results in enhanced risks or interferes with the intervention [12], it is possible that medications could influence motor performance in the proposed task. Future research is necessary to explore this putative interaction and provide a more comprehensive understanding of its impact. (3) Although psychological factors are known to impact motor function and overall treatment outcomes, we did not analyze anxiety and depression, and their omission may have influenced the results. Future studies should incorporate these assessments in order to better understand the interplay between psychological well-being and motor performance improvements. (4) The control group received sham tDCS but still participated in VR tasks, which might have therapeutic effects on its own. A control group with no intervention or a different form of therapy could provide a clearer comparison. (5) The present study focused on improving motor performance in women with fibromyalgia; therefore, we did not assess pain levels, as this was not the primary aim of the study. However, given the central role of pain in fibromyalgia, its omission limits our understanding of the intervention’s full benefits. Future studies should include a pain assessment to evaluate the potential analgesic effects of tDCS and VR therapy.

## 5. Conclusions

In conclusion, this study demonstrates that using transcranial direct current stimulation during virtual reality tasks significantly improves motor performance in women with fibromyalgia. Participants who received active tDCS showed lasting improvements in motor function, with increased hits and reduced absolute and variable errors, especially in tasks requiring more complex movements. The results indicate that tDCS can effectively complement VR-based rehabilitation strategies. Additionally, VR tasks, through real-time feedback and repeated practice, proved to be a valuable tool for enhancing motor skills, even when the tDCS effects were minimal, highlighting its importance in fibromyalgia management.

## Figures and Tables

**Table 1:** Characterization variables between groups.

	GA	GB	
	Mean ± Standard Deviation	Mean ± Standard Deviation	*p*-Value
Age (years)	41.4 (6.7)	41.3 (7.0)	0.820
Height (m)	1.61 (0.05)	1.60 (0.03)	0.121
Weight (Kg)	81.4 (20.4)	68.7 (10.9)	0.305
Time of diagnosis (years)	7.75 (5.49)	5.16 (3.65)	0.214
Comorbidities	n (%)	n (%)	
Hypertension	2 (9.5)	1 (4.7)	0.538
Depression	1 (4.7)	0 (0)	0.524
Asthma	1 (4.7)	0 (0)	0.524
Use of technology			
Experience with computer	9 (42.9)	3 (14.3)	0.538
Experience with games	9 (42.9)	3 (14.3)	0.633

GA: Group A (tDCS active-sham); GB: Group B (tDCS sham-active); Kg: kilograms; m: meters. Significance of the continuous data determined through the Mann–Whitney U test and for categorical data through the chi-square test.

**Table 2:** Description of medications taken by the subjects of the study, by category.

Subject	Antihypertensives	Thyroid Hormones	Antidepressants	Muscle Relaxants	Analgesics	Vitamins and Supplements	Anti-Allergic/Respiratory	Antiseizure
1	Olmesartan/Hydrochlorothiazide, Metoprolol		Daforin					
2	Olmesartan/Hydrochlorothiazide, Metoprolol	Euthyrox	Escitalpram			Coenzime Q10		
3			Amitriptyline	Cyclobenzaprine				
4			Duloxetine		Pregabalin	Calcium, Vitamin D		
5								
6								
7			Duloxetine				Montelukast	
8							Beclomethasone, Pizotifen	
9			Nortriptyline, Duloxetine		Pregabalin			
10			Amitriptyline	Cyclobenzaprine				
11								
12		Euthyrox				Calcium, Carbonate, Calcitriol, Vitamin D		
13								
14								
15								
16	Olmesartan/Hydrochlorothiazide, Metoprolol			Cyclobenzaprine				
17			Nortriptyline, Duloxetine		Tramadol			
18			Duloxetine		Pregabalin			
19			Clonazepam					Topamirate
20								
21								

## Data Availability

The raw data supporting the conclusions of this article will be made available by the authors on request.
